# Klippel-Feil Syndrome and Unilateral Diaphragmatic Paralysis

**Published:** 2015-02-10

**Authors:** John Cece, Farid Aboharb, Kameron S. Rezzadeh, David Brown, Reza Jarrahy, Matthew R. Kaufman

**Affiliations:** ^a^The Institute for Advanced Reconstruction, Shrewsbury, NJ; ^b^Department of Rehabilitation Medicine, JFK Medical Center, Edison, NJ; ^c^Division of Plastic and Reconstructive Surgery, Department of Surgery, David Geffen School of Medicine at UCLA, Los Angeles, Calif.

**Keywords:** Klippel-Feil, phrenic nerve, nerve transfer, cervical, spinal cord

## DESCRIPTION

A 12-year-old boy with known Klippel-Feil Syndrome involving fusion of the C5-C6 vertebrae presented to the clinic complaining of a 4-year history of dyspnea on exertion. The patient was referred to our practice following multiple unsuccessful bronchodilator treatments for the presumptive diagnosis of asthma. The patient's medical history included multiple hospital admissions at a different institution for critical respiratory tract infections during infancy. Of note, fluoroscopic evaluation of the patient during those admissions did not reveal diaphragmatic paralysis.

## QUESTIONS

1. **What is Klippel-Feil Syndrome and how is it classified?**
2. **What are the extraspinal manifestations of this condition?**
3. **How was the diagnosis of unilateral diaphragmatic paralysis confirmed in this patient?**
4. **Describe how diaphragmatic paralysis was treated in this patient as well as the postoperative course.**

## DISCUSSION

Klippel-Feil Syndrome (KFS) is a congenital skeletal disorder characterized by a defect in cervical spine segmentation leading to various degrees of pathologic vertebral fusion in affected patients.^1^^,^^2^ Klippel-Feil Syndrome is classically associated with a low posterior hairline, short neck, and limited cervical range of motion. Although these features were initially considered to be the diagnostic “triad” of KFS, we now know that fewer than 50% of KFS patients present with all 3 of these findings.^3^^,^^4^ Klippel-Feil Syndrome patients are affected within a broad range of clinical severity and functional disability. The current KFS classification scheme is based on extent of cervical spine involvement as well as extracervical manifestations. Scoliosis, Sprengel's deformity, and cervical ribs are among the bony anomalies with a well-documented association with KFS.^1^

Klippel-Feil Syndrome is a physically debilitating and psychologically devastating congenital skeletal abnormality whose extraspinal manifestations remain poorly understood. More recent studies on KFS suggest an emerging awareness of a diverse variety of extraspinal manifestations of this disease including genitourinary, cardiovascular, and neurological sequelae.^1^ Although respiratory distress is a frequent complaint among patients with extensive KFS disease whose respiratory mechanics are affected by thoracic deformity, KFS has not been associated with respiratory symptoms as a result of phrenic nerve dysgenesis prior to this case report.

Together, clinical history and examination findings supported an incomplete phrenic nerve lesion leading to right hemidiaphragmatic paralysis. Intraoperatively, the patient was found to have an atrophic phrenic nerve arising solely from the C5 nerve root, without the expected adjacent contributions from C3 or C4. Dense adhesions (consistent with those resulting from compression neuropathy) were observed around both the C5 nerve root and the atrophic right phrenic nerve. These adhesions were most prominent in the proximal region of the nerve. An atrophic phrenic nerve fed by only the C5 nerve root is a novel finding in a patient with KFS. Direct nerve stimulation of both the C5 nerve root and phrenic nerve revealed intact but diminished activity in the diaphragm, requiring supraphysiologic threshold amplitudes (>3 mA) to elicit a response.

Phrenic nerve reconstruction, including nerve decompression and transfer, was used to treat this patient. Following identification of the spinal accessory nerve, a nerve transfer was performed without nerve sacrifice. The patient began to experience symptomatic relief in the early postoperative period (1-2 weeks) with signs of improvement on radiographic imaging. On examination 1 year after his surgery, the patient's functional improvement was confirmed by postoperative electrodiagnostic and spirometric testing, which suggested enhanced nerve conduction and ventilation, respectively.

The pathophysiology of acquired phrenic nerve dysgenesis in KFS is unclear, but we hypothesize that this may have occurred as a result of the skeletal abnormalities associated with the KFS phenotype. Phrenic nerve reconstruction for restoration of functional activity in the diaphragm has been expanded by the senior author (M.K.), and a recently published cohort analysis to evaluate outcomes of phrenic nerve reconstruction for diaphragmatic paralysis includes an updated treatment algorithm.^5^ Future work leading to the elucidation of other extraspinal manifestations of KFS may be beneficial in determining the pathophysiology of this clinically heterogeneous and poorly understood condition.

## Figures and Tables

**Figure 1:**
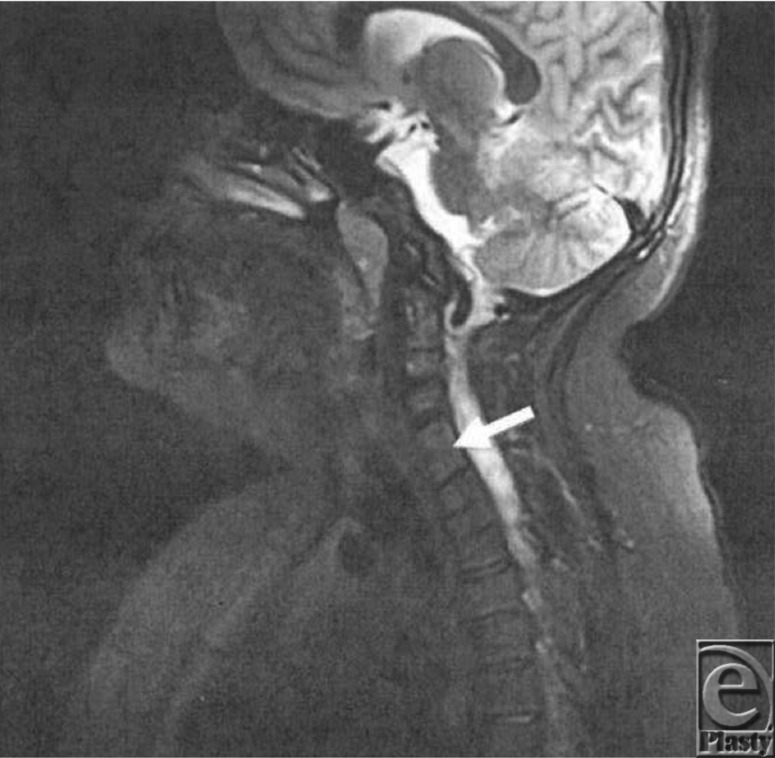
Sagittal plane, T2-weighted sequence with contrast showing segmentation anomalies of cervical vertebrae.

**Figure 2:**
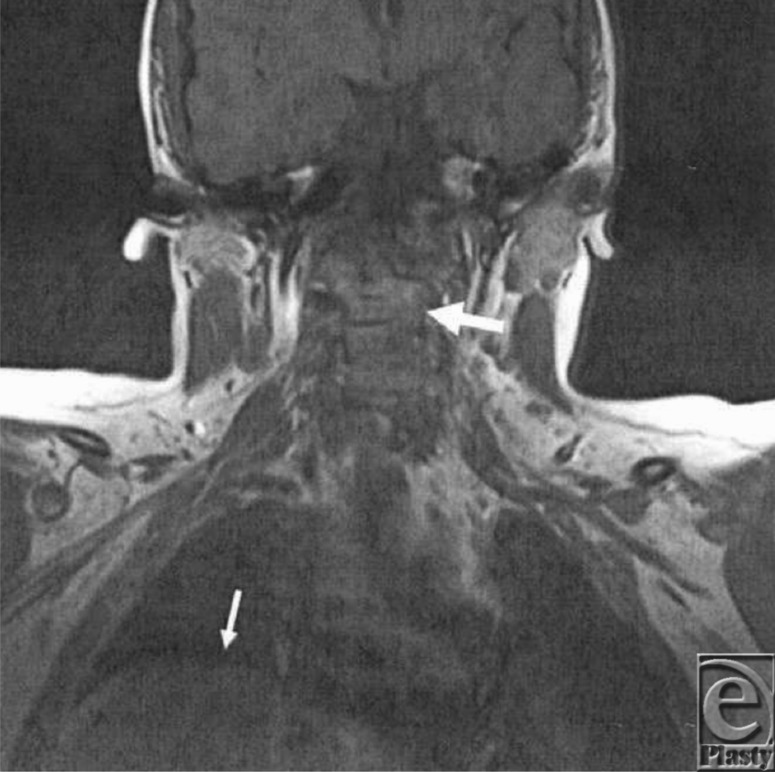
Coronal plane, T1-weighted sequence before surgery showing the fused cervical vertebrae (big arrow). We also noted elevation of the right hemidiaphragm (small arrow) indicative of unilateral diaphragmatic paralysis.
